# Supplementary material for: Factors Impacting Retention of Aged Care Workers: A Systematic Review
Source: Healthcare (Basel). 2023 Nov 21;11(23):3008. doi: 10.3390/healthcare11233008 (PMC10706301; doi:10.3390/healthcare11233008)
Supplement: Supplementary file 1 [file healthcare-11-03008-s001.zip › Table S2 ENTREQ statement.pdf]

Table S2. Enhancing transparency in reporting the synthesis of qualitative research: the ENTREQ statement

No	Item	Description	Examples/location in manuscript
1	Aim	What research question does the synthesis address?	Barriers and facilitators to retention of aged care workers
2	Synthesis methodology	What synthesis methodology or theoretical framework underpins the synthesis? Is a rationale for choice of method provided?	JBI mixed methods review methodology
3	Approach to searching	Was the search pre-planned or iterative?	pre-planned
4	Inclusion criteria	Were inclusion/exclusion criteria provided	Table 1
5	Data sources	Are all the information sources and the date of searches stated? Is a rationale provided?	Section 2.1
6	Electronic search strategy	Were details of a search strategy provided?	Table S3
7	Study screening methods	Were the study screening and sifting process described?	Section 2.2
8	Study characteristics	Were the characteristics of the included studies presented?	Table 2 and tables S4-S6
9	Study selection results	Numbers of studies screened and reasons for exclusion indicated in a figure/flowchart	Figure 1 PRISMA flowchart
10	Rationale for appraisal	What was the rationale and approach used to appraise the included studies or selected findings?	Section 2.3
11	Appraisal items	What tools, frameworks and criteria were used to appraise the studies or selected findings?	Section 2.3
12	Appraisal process	Who conducted the appraisal?	Section 2.3
13	Appraisal results	Were the results of the quality assessment presented? Were articles weighted/excluded based on assessment and was this justified?	Table 1; section 2.3
14	Data extraction	How were the data extracted from the primary studies?	Section 2.4
15	Software	What computer software, if applicable, was used?	Section 2.4
16	Number of reviewers	Who was involved in coding and analysis?	Section 2.4
17	Coding	Was the process for coding of data reported?	Section 2.4
18	Study comparison	How were comparisons made within and across studies?	Section 2.4
19	Derivation of themes	How were the themes or constructs derived?	Section 2.4, section 3.4

20	Quotations	Were quotations from the primary studies provided to illustrate themes/constructs?	No
21	Synthesis output	Did the synthesis generate rich, compelling and useful output that went beyond a summary of the primary studies?	Section 3.5-3.8, Figure 2
