# Supplementary material for: Factors Impacting Retention of Aged Care Workers: A Systematic Review
Source: Healthcare (Basel). 2023 Nov 21;11(23):3008. doi: 10.3390/healthcare11233008 (PMC10706301; doi:10.3390/healthcare11233008)
Supplement: Supplementary file 1 [file healthcare-11-03008-s001.zip › Table S3_Medline search strategy.pdf]

Table S3. Medline search strategy

Search ID#	Search Terms	Search Notes
S1	"nurs* assistan*".mp.	
S2	"nurs* aide*".mp.	
S3	"personal care*".mp.	
S4	"disability support".mp.	
S5	"frontline care*".mp.	
S6	"geriatric nurs*".mp.	
S7	"care support work*".mp.	
S8	Geriatric Nursing/	MeSH term
S9	"Hospice and Palliative Care Nursing"/	MeSH term
S10	Nursing Assistants/	MeSH term
S11	1 or 2 or 3 or 4 or 5 or 6 or 7 or 8 or 9 or 10	
S12	"nursing home*".mp.	
S13	"long-term care".mp.	
S14	"hospice*".mp.	
S15	"residential facilit*".mp.	
S16	"homes for the aged".mp.	
S17	"assisted living".mp.	
S18	"aged care".mp.	
S19	"home-based care".mp.	
S20	Homes for the Aged/	
S21	Nursing Homes/	MeSH term
S22	12 or 13 or 14 or 15 or 16 or 17 or 18 or 19 or 20 or 21	
S24	S11 and 22	

S25	"barrier*".mp.	
S26	"facilitator*".mp.	
S27	"job satisfaction".mp.	
S28	"attitude*".mp.	
S29	"recruitment".mp.	
S30	"retention".mp.	
S31	"professional development".mp.	
S32	"motivation".mp.	
S33	"challenge*".mp.	
S34	"employment".mp.	
S35	25 or 26 or 27 or 28 or 29 or 30 or 31 or 32 or 33 or 34	
S36	12 and 23 and 35	
S37	limit 36 to yr="2012 -Current"	

[mp=title, book title, abstract, original title, name of substance word, subject heading word, floating sub-heading word, keyword heading word, organism supplementary concept word, protocol supplementary concept word, rare disease supplementary concept word, unique identifier, synonyms, population supplementary concept word, anatomy supplementary concept word]
