# Supplementary material for: Factors Impacting Retention of Aged Care Workers: A Systematic Review
Source: Healthcare (Basel). 2023 Nov 21;11(23):3008. doi: 10.3390/healthcare11233008 (PMC10706301; doi:10.3390/healthcare11233008)
Supplement: Supplementary file 1 [file healthcare-11-03008-s001.zip › Table S4_Details of interventional studies.pdf]

Table S4. Details of quantitative interventional studies

Author	Sampling	Intervention<br><br>(Abbreviated TIDieR checklist)	[a]Care support worker demographics	Outcomes	Results
Jeon (2015)[45]	Convenience sampling from 45 sites within a single aged care organisation across both urban and rural areas. 24 sites randomly selected	*Name:* Clinical Leadership in Aged Care (CLiAC)<br>*Why:* To develop leadership and management skills in middle managers<br>*What*: 12-month leadership training program, incorporating action learning techniques, feedback, case scenarios and individual practice improvement projects<br>*Who:* A facilitator with extensive nurse manager experience<br>*How:* Individual meetings every four to six weeks, group discussions and workshops supplemented by learning resources<br>Control group: usual education and training	Gender: NR<br>Age: Intervention group 14% <35, 25% 35-44, 35% 45-54, 26% >55 years;<br>Control group 18% <35, 16% 35-44, 37% 45-54, 29% >55 years<br>Employed: Intervention group 30% <3, 19% 3-5, 35% 5-10, 16% >10 years;<br>Control group 25% <3, 12% 3-5, 33% 5-10, 30% >10 years	Work environment<br>Care quality and safety<br>Work stress<br>Staff turnover rates<br>Staff stress level<br>Staff absenteeism	Work environment: Significant difference favouring intervention in greater supervisor support (0.61, 95% CI 0.04-1.18, p =0.04), perception of manager leadership style and behaviours (0.30, 95% CI 0.09-0.51, p =0.005) and leadership outcomes (0.35, 95% CI 0.13-0.56, p=0.001)<br>Care and quality: No difference in care staff attitudes towards dementia care or person-centred care.<br>Staff turnover: No effect of the intervention (p=0.16)<br>Care staff job satisfaction, stress, and intention to leave: No effect of the intervention
Ericson-Lindman (2017)[46]	Random selection of a single RACF from 21 potential facilities in an urban area	*Name:* Not reported<br>*Why:* To address stress of conscience in aged care staff<br>*What*: Participatory action research intervention<br>*Who:* Not reported<br>*How:* 12 sessions of 90-minute duration with RN and NA participants together, using problem-based learning to learn how to constructively deal with issues of troubled conscience.	Gender: 89% female<br>Age: 52 (7.4) years<br>Employed: 22 (7.6) years	Stress of conscience<br>Perception of conscience<br>Burnout<br>Social support	Perception of conscience: Significantly less agreement with "Our conscience warns us against hurting others" (p=0.04)<br>Social support: Significantly higher score "Are your work achievements appreciated by your immediate superior?" (p<0.001)<br>Stress of conscience and burnout: No significant difference
O'Brien (2019)[47]	Convenience sampling from "several" LTCFs in an urban area	*Name:* Not reported<br>*Why:* To understand the experience of care staff undergoing group therapy<br>*What*: Acceptance and Commitment Therapy (ACT) focused on workplace	Gender: 86% female<br>Age: 37.9 (13.2) years<br>Employed: 12 (9.9) years	Workplace injuries-days absent, frequency, injury complaint<br>Mental Health	Days missed due to injury: Significantly less days missed for treatment group at 1-month follow up, F (1, 57) = 7.59, p = 0.008, n^2 = 0.12

	challenges and stressors and methods to constructively deal with them<br>*Who:* Not reported<br>*How:* Two 2.5-h sessions of group-based therapy spaced 1 week apart	Group therapy process	Injury frequency or complaints: nil difference within or between-groups<br>Mental health: Significant reduction for treatment group in mental health symptoms F (1, 28) = 9.28, p = 0.005.<br>Themes from process evaluation:<br>Mindfulness exercises an interesting way to reconceptualize challenges at work.<br>Group-based therapy well-received and encouraged greater team bonds through common experiences

[a] Data presented as mean (SD) where possible, converted using standard calculations where required. ACA, aged care assistant; AIN, assistant in nursing; CA, care aide; CNA, certified nursing assistant; DCW, direct care workers; DON, director of nursing; LN, licensed nurse; HHA, home health aide; LTCF, long-term care facility; n/a, not applicable; NA, nurse assistant; NH, nursing home; NR, not reported; PCW, personal care worker; PSW, personal support worker; RACF, residential aged care facility; RN, registered nurse; SNF, skilled nursing facility
