# Supplementary material for: Factors Impacting Retention of Aged Care Workers: A Systematic Review
Source: Healthcare (Basel). 2023 Nov 21;11(23):3008. doi: 10.3390/healthcare11233008 (PMC10706301; doi:10.3390/healthcare11233008)
Supplement: Supplementary file 1 [file healthcare-11-03008-s001.zip › Table S5_Details of quantitative and mixed methods descriptive studies.pdf]

Table S5. Details of quantitative and mixed methods descriptive studies

Author	Sampling	[a]Care support worker demographics	Outcomes	Results
Berridge (2022)[81]	Nationwide purposive sampling from 2124 NHs who participated in a 2008-2009 survey that were still operational in 2016	Gender: n/a<br>Age: n/a<br>Employed: n/a	Association of retention and leadership/staff empowerment and nursing home covariates-profit status, facility size, occupancy rate, nursing ratios and local competition for staffing	Leadership and staff empowerment score most significantly associated with NA retention, with RRR of retention 76% to 90% relative to 0% to 50% retention 2.81 (CI: 1.73-4.58).<br>Higher NH administrator turnover (3 or more in past year) was associated with lower NA retention (0%-50%, RRR = 0.62, CI:0.41-0.93).<br>Higher NH occupancy significantly associated with greater NA retention of 75% to 90% (RRR = 1.08, CI: 1.01-1.15)
Beynon (2022)[88]	Purposive sampling from 4 NHs	Gender: 78% female<br>Age: 33 (13.52) years<br>Employed: 9 (8.8) years	Team collaboration<br>Experience of collaborating as pairs of nurses/CNAs	From survey results (Aim 1), there was no significant difference across the facilities between LN and CNA perception of teamwork and collaboration and reflected a collaborative environment between staff with differing roles.<br>Exploration of lived work experience (Aim 2) identified five interconnected themes of perspective (attitudes participants bring to work), co-worker connection (getting to know colleagues as people), communication (sharing of ideas and observations), mutual support (helping each other) and "it makes a difference" (regarding impact of collaboration)
Brown (2016)[90]	Convenience sampling from 11 US states with Green House NHs and comparison NHs with random selection of final yield	Gender: Green House 91% female; NH 84% female<br>Age: Green House 38.8 (11.5); NH 37.2 (10.9) years<br>Employed: Green House 4.1 (4.6) years; NH 3.8 (3.8) years	Work stress<br>Safety culture<br>Staff satisfaction<br>Staff hours to resident ratios<br>Staff turnover	Satisfaction (CNAs): Only significant difference related to difficulty of job in presence of absenteeism; GH 58.2%, NH 80.5%, $p<0.05$<br>Turnover (CNAs): No significant difference between sites; GH 47.4 (46.1), comparison NH 60.5 (28.7).<br>Safety culture, staff ratios and work stress no significant difference for CNAs between sites
Chang (2021)[70]	Purposive sampling of all LTCFs in urban region	Gender: 84% female<br>Age: 21% aged 30-39 years, 21% 40-49 years, 28% 50-59 years, 13% >60 years<br>Employed: 26% 5-10 years, 22% >10 years, average 7.5 years	Job competency<br>Job satisfaction<br>Intention to stay	NA job competency had a direct positive effect on intrinsic satisfaction (point estimate 0.345, 95% CI 0.584, 0.715, $p<0.001$), and extrinsic job satisfaction (point estimate 0.488, 95% CI 0.410, 0.584, $p<0.001$) and a mediating effect on intention to stay via both intrinsic and extrinsic satisfaction ($p<0.05$)

Chao (2020)[80]	Nationwide stratified equal probability sampling	Gender: "Younger" 77% female; "Older" 87% female<br>Age: "Younger" 36.15 (6.9), "Older" 54.42 (5.3) years<br>Employed: NR	Personal characteristics<br>Organisational support<br>Work latitude<br>Selection, optimization, and compensation<br>Burnout<br>Intention to stay<br>Retention	Older NAs reported significantly lower perceived support ($t = 1.99$, $p<0.05$), less emotional exhaustion/burnout (t = 3.84, $p<0.001$), increased ability to prioritize goals (t = -2.813, $p<0.01$), greater personal accomplishment (t = -3.46, $p<0.01$), and a higher intention to stay when income was higher ($b = 0.19$, $p< 0.05$).<br>Retention of older NAs was significantly influenced by work latitude, optimization strategy, and emotional exhaustion
Charlesworth (2020)[49]	Nationwide purposive sampling of all RACFs and home care providers	Gender: HCW Australian born 91.2% female, ESB 89.3% female, NESB 87.2% female; PCA Australian born 90.2%, ESB 91.8% female, NESB 80.3% female<br>Age: HCW Australian born 50.4 years, ESB 52.7 years, NESB 49.1 years; PCA Australian born 45.4 years, ESB 48.5 years, NESB 40.8 years<br>Employed: HCW Australian born 6.4 years, ESB 6.0 years, NESB 5.8 years; PCA Australian born 6.8 years, ESB 6.3 years, NESB 5.8 years	Personal characteristics<br>Years in Australia<br>Residency status<br>Highest post-school qualification<br>Employment contract<br>Length of tenure<br>Preferred hours	Migrant HCWs and PCAs are significantly more likely have a higher degree qualification (OR = 0.15, $p<0.001$), be employed on causal contracts (OR = 18.15, $p<0.001$), and desire more weekly hours of work than Australian-born peers (OR = 1.95, $p<0.001$). Being from a NESB rather than a migrant from an ESB increased the odds of being in casual employment (OR = 0.96, $p<0.001$)
Cheung (2018)[78]	Nationwide convenience sampling with 365 NHs contacted	Gender: 98% female<br>Age: 51.1 (9.6) years<br>Employed: 10.4 (6.4) years	Musculoskeletal symptoms<br>Perceived physical exertion<br>Response to work demands<br>Ergonomic and manual handling knowledge<br>Job content, satisfaction, and stress<br>Intention to leave	Prevalence of symptoms: 88.4% reporting at least one current musculoskeletal injury. This was significantly associated with female gender (98.5%, $p=0.04$), perception of health status to be "not good" (49.4%, $p<0.001$), greater work stress (72.4%, $p<0.0001$) and intention to leave (37.3%, $p= 0.013$).
Dhakal (2020)[85]	Purposive sampling of 11 facilities in single	Gender: 89% female<br>Age: 54% > 40 years	Work environment<br>Intention to leave	Intention to leave significantly associated with casual workers, workers <39 years old, those working in metropolitan areas ($p<0.001$). Factors

	state from same organisation	Employed: 33% < 2, 24% >11 years	Perceived attributes of ACAs	affecting intention to leave included heavier workloads, limited teamwork, low staffing. Retention associated with pay rates, available working hours, promotion opportunities and job security
Dys (2022)[92]	Stratified purposive sampling across rural and urban areas with 93 NHs contacted	Gender: 79% female<br>Age: DCW 35.7 (12.7) years<br>Employed: 5.5 (7.3) years	Organisational characteristics<br>Person centred care practices<br>Job Satisfaction<br>Affective commitment<br>Intention to leave	No significant difference between DCWs and nurse intention to leave, job satisfaction, affective commitment, or perceptions of "knowing the person" and "comfort care."
Graham (2012)[61]	Purposive sampling from public list of all CNAs within a US state	Gender: 91% female<br>Age: 35 (12) years<br>Employed: 51% 1-5, 6% 6-10, 11% 11-15, 11% 16-20, 6% 21-25, 11% >25 years	Prevalence of work-related injury<br>Work-tasks related to injury<br>Work-related manual handling training<br>Perception of work	Injury prevalence: 46% reporting experience of work-related injury, 40% of these reported a back injury with 86% of these occurring when moving or lifting a patient.<br>Work experience: 69% reported injury had been in role > 5 years<br>Training: Those who reported "a whole lot of training, 5/5" accounted for 79% of those with back injury incidence<br>"Hardest part of job": Key themes identified as too many patients; not enough help to care for patients; and poor relationships between nurses and CNAs
Isherwood (2017)[54]	Nationwide purposive sampling of all RACFs and home care providers	Gender: PCA Australian born 90.2%, ESB 91.8% female, NESB 80.3% female<br>Age: PCA Australian born 45.4 years, ESB 48.5 years, NESB 40.8 years<br>Employed: PCA Australian born 6.8 years, ESB 6.3 years, NESB 5.8 years	Socio-demographic<br>Work arrangements<br>Job satisfaction<br>Retention and intention to leave	South-East Asian-born migrant care workers found to have significantly higher rates of Bachelor degree qualification (35.6%, $p<0.001$) than locally born peers (2.4%). North-east Asian migrant workers reported significantly higher levels of an expectation to work longer than scheduled hours (21.6%, p<0.001) compared to locally born peers (11.4%). Asian-born care workers reported higher job satisfaction across all areas including respect from others, total pay, development opportunities and time with care recipients than local-born peers ($p<0.001$). South-east Asian born employees were more likely to be actively looking for new employment (13.3%, $p<0.001$) however intention to leave over the next three years was not different between any Asian born or locally born groups
Keisu (2018)[93]	Purposive sampling from respondents to previous survey in urban and rural areas, nine workplaces	Gender: n/a<br>Age: n/a<br>Employed: n/a	Effort and reward<br>Transformational leadership	NAs reported significantly greater effort scores 18.7 (5.2), p<0.001). No significant difference in reward scores between NAs and professionals. Professionals scored significantly higher on positive assessment of managers being transformational leaders (25.4% vs 13.5%, $p=0.026$) and charismatic role models (36% vs 9%, $p=0.001$)

	strategically selected from 132			
Kennedy (2020)[82]	Purposive sampling from 835 NHs with complete retention and turnover data from a single US state (rural and urban)	Gender: n/a<br>Age: n/a<br>Employed: n/a	Turnover<br>Retention<br>Covariates including organisational structure, resident case mix, financial resources, staffing and management	Average CNA turnover 54.7%; average CNA retention rate 63.8%. Turnover and retention significantly negatively correlated (Pearson correlation, r = -0.26, $p<0.001$).<br>Higher CNA turnover associated with for-profit ownership status ($b=6.83$ (3.08), $p<0.05$), higher DON turnover ($b=8.28$ (3.64), $p<0.05$), higher proportion of residents with psychiatric illness ($b=0.18$, $p<0.01$) and low local unemployment rates ($b=-3.81$ (1.46), $p<0.05$)
Leskovic (2020)[91]	Purposive sampling from 86 NH in 2013 and 98 NH in 2020 from rural areas	Gender: 96% female<br>Age: 40.8 (9.6) years<br>Employed: NR	Burnout<br>Job satisfaction<br>Experience of occupational changes during the pandemic (2020 only)	Burnout: Significantly greater emotional exhaustion ($p<0.001$) and significantly less personal accomplishment ($p<0.001$) in 2020 than 2013.<br>Satisfaction: Significantly less intrinsic ($p=0.01$), extrinsic ($p=0.004$) and overall satisfaction ($p=0.028$) in 2020 than 2013.<br>Experience of working during a pandemic: Themes included consistent experience of physical, emotional, cognitive, and behavioural burnout, however greater engagement with co-workers and residents. Job satisfaction was negatively impacted by direct work environment changes to work hours and lack of breaks, but was bolstered by a sense of cooperation between staff
Liang (2014)[52]	Proportional stratified random sampling of LTCFs	Gender: NR<br>Age: 43.1 (10.9) years<br>Employed: NR	Job stress<br>Health-related quality of life	Physical quality of life significantly positively associated with longer shift length (12 hours vs 8 hours (t=2.650, $p< 0.01$) and negatively associated with higher psychological demand (t= -5.699, $p< 0.01$).<br>Mental quality of life significantly positively associated with increased worker age (t=3.525, $p< 0.01$), smaller institution size (t=2.802, $p< 0.01$), and increased job control (t=3.460, $p< 0.01$) and significantly negatively associated with (t= -4.496, $p< 0.01$)
Lin (2021)[60]	Convenience sampling from 12 LTCFs across a region	Gender: 87% female<br>Age: 30% <50, 33% 51-55, 37% >55 years<br>Employed: 33% 1-2, 35% 3-5, 31% >6 years	Socio-demographics<br>Work experience<br>Training<br>Intention to leave<br>Supportive supervision	Increased years of working as a PSW, nurse supervisor gender (female), higher education, increased number of years in nursing, presence of nurse management training and higher RN/PSW ratio all positively significantly influenced the level of supportive supervision reported by the PSWs (all $p<0.05$).
Matthews (2018)[94]	Purposive sampling from 22 LTCFs across single state	Gender: n/a<br>Age: n/a<br>Employed: n/a	Employee perceptions of leadership<br>Turnover<br>Job satisfaction<br>Time spent with supervisor	Retained employees had significantly greater loyalty, affect, respect and contribution for their supervisor than staff who had turned over (all p<0.001). There was no significant difference between retained or staff who had turned over with relation to job satisfaction, age or hours interacting with supervisor per week.

				When controlling for job satisfaction, age, gender, and time interacting with supervisor, only supervisor loyalty was significantly associated with staff turnover (pseudo R^2=0.16, *p*<0.01)
Rodríguez-Monforte (2020)[73]	Purposive sampling from 364 facilities	Gender: 94% female<br>Age: 45.3 (10.4) years<br>Employed: 7.6 (6.7) years	Job satisfaction<br>Work empowerment<br>Work effectiveness<br>Work stress<br>Supervisory support	Job satisfaction of NAs was significantly negatively associated with stress from resident behaviour (*b*=-0.20 95%CI -0.32, -0.07, *p*<0.01), lower supervisory support (*b*=0.16 95%CI 0.12, -0.21, *p*<0.01), lower work effectiveness (*b*=0.14 95%CI 0.10, 0.18, *p*<0.01) and lower work empowerment (*b*=-0.22 95%CI 0.14, 0.29, *p*<0.01)
Rodwell (2015)[83]	Convenience sampling	Gender: 86% female<br>Age: >58% older than 50 years<br>Employed: 50% < 1 year, 38% 1-4 years	Workplace bullying<br>Workplace violence<br>Job demand<br>Social support<br>Negative affectivity	Experiencing bullying by CNAs was significantly positively related to higher negative affect (0.36, *p*<0.05), working part-time compared to casually (3.35, *p*<0.01) and lower outside social support (0.35, *p*<0.05). Higher workload was significantly positively associated with higher physical assaults from resident's families (external abuse) (0.09, *p*<0.05), and increased age of worker was associated with significantly less reports of assault (-2.47, *p*<0.001) or threat<br>(-1.2, *p*<0.05) from resident's families
Sharma (2022)[48]	Purposive sampling of 439 NHs from single state	Gender: n/a<br>Age: n/a<br><br>Employed: n/a	Turnover<br>Average hourly wage<br>Resident and facility characteristics	Higher wages significantly associated with lower turnover of CNA staff and this was more prominent in for-profit facilities. Covariates of occupancy, age, ethnicity, or gender of residents, staffing hours per day, local unemployment rates or facility location did not affect turnover rates of CNAs in this analysis
Stevens (2022)[51]	Purposive sampling from 83 NHs in urban region	Gender: 96% female<br>Age: 45.4 (10.5)<br>Employed: NR	Quantitative work demands<br>Psychosocial work environment<br>Step rates per hour<br>Observations of direct care tasks	Lower perceived quantitative demands at work were associated with being a migrant (-6.5, 95%CI -11.0, -2.0, *p*=0.006), being a care helper compared to care aide (-8.1, 95%CI -11.9, -4.1, *p*<0.001), working a fixed evening (-16.6, 95%CI -24.7, -8.6, *p*<0.001), or evening/night shift (-22.7, 95%CI -41.4, -3.7 *p*=0.021), having less emotional demands at work (0.3, 95%CI 0.2, 0.4, *p*<0.001), and having less influence at work (-0.2, 95%CI -0.3, -0.1, *p*<0.001)
Wallin (2012)[72]	Purposive sampling across three urban districts	Gender: 86% female<br>Age: 44.1 (11.0) years<br>Employed: 16.5 (9.8) years	General job satisfaction<br>Satisfaction with nursing-care provision<br>Health complaints<br>Sense of coherence<br>Personalised care<br>Work climate<br>Quality of care<br>Leadership and support	General job satisfaction was significantly negatively associated with NAs reporting health complaints, and significantly positively associated with the caring climate and personalised care provision. Nursing-care satisfaction was significantly positively associated with married NAs, those working day shifts, and the caring climate and personalised care provision

Yang (2021)[53]	Purposive sampling from across urban and rural areas	Gender: 90% female<br>Age: 44 (13.6) years<br>Employed: 6.5 (5.9) years	Job content<br>Musculoskeletal pain	Job control and social support were significantly negatively associated with musculoskeletal pain ($p<0.001$), with lower social support correlating with the more severe pain of the NA ($p<0.001$). Pain scores were significantly higher for NAs with chronic disease, those without exercise habits, those without rest time at work, those who didn't use assistive devices, those who were migrants and those of older age ($p<0.001$)
Zhang (2016)[75]	Convenience sampling from 15 non-unionised facilities	Gender: 93% female<br>Age: 40.5 (13.7) years<br>Employed: NR	Mental health<br>Work-family conflict<br>Sleep quantity/quality<br>Working conditions	Higher levels of work-family conflict were significantly positively correlated with lower mental health scores (Rho = −0.16, $p< .01$), increased physical demands, psychological demands, and weekly work hours ($p< .05$). Decision latitude, social support, and shift length were significantly negatively correlated with work-family conflict ($p< .05$)

[a] Data presented as mean (SD) where possible, converted using standard calculations where required. ACA, aged care assistant; CA, care aide; CNA, certified nursing assistant; DCW, direct care workers; DON, director of nursing; ESB, English speaking background; HCW, home care worker; HHA, home health aide; LN, licensed nurse; LTCF, long-term care facility; n/a, not applicable; NA, nurse assistant; NESB, non-English speaking background; NH, nursing home; NR, not reported; PCW, personal care worker; PSW, personal support worker; RACF, residential aged care facility; RN, registered nurse; RRR, relative risk ratio; SNF, skilled nursing facility
