# Supplementary material for: Factors Impacting Retention of Aged Care Workers: A Systematic Review
Source: Healthcare (Basel). 2023 Nov 21;11(23):3008. doi: 10.3390/healthcare11233008 (PMC10706301; doi:10.3390/healthcare11233008)
Supplement: Supplementary file 1 [file healthcare-11-03008-s001.zip › Table S6_Details of qualitative studies.pdf]

Table S6. Details of qualitative studies

Author	Sampling	Care support worker demographics	Data analysis
Adebayo (2023) [55]	Purposive sampling from RACFs, ethnic groups and multicultural support services across urban areas	Gender: 75% female<br>Age: 25-45 years<br>Employed: 1-10 years	Thematic analysis
Amateau (2023)[64]	Convenience sampling to recruit nursing facilities and staff from urban, suburban, and rural areas	Gender: 94% female<br>Age: 41.0 (12.6) years<br>Employed: 15 years	Thematic analysis
Bergqvist (2022)[76]	Purposive sampling from 318 NHs in single region	Gender: 95% female<br>Age: Average 51-60 years<br>Employed: >5 years	Thematic analysis
Booi (2021)[65]	Purposive sampling from single LTCF in rural area	Gender: 84% female<br>Age: 42.0 (7.0) years<br>Employed: 9 (7.5) years	Thematic analysis
Cooke (2021)[87]	Purposive sampling from two suburban LTCFs	Gender: 100% female<br>Age: 44.0 (10.5)<br>Employed: 10.6 years	Thematic analysis
Creapeau (2022)[62]	Purposive sampling from 59 urban and rural SNFs	Gender: CNAs 77% female<br>Age: CNAs 15% <22, 27% 23-30, 24% 31-40, 14% 41-50, 20% >51 years<br>Employed: CNAs 43% <2, 19% 2-3, 17% 4-7, 15% 8-20, 6% >21 years	Content analysis
Dijxhoorn (2023)[89]	Nationwide convenience sampling	Gender: 94% female<br>Age: 48.9 (11.4)<br>Employed: 29% <10, 24% 10-25, 47% >25 years	Thematic analysis
Franzosa (2022)[77]	Purposive sampling from 289 LTCFs	Gender: CNAs 95% female<br>Age: 23% 20-39, 59% 40-59, 16% >60 years<br>Employed: 7% 0-3, 10% 3-5, 21% 5-10, 59% >10 years	Content analysis
Gleason (2016)[58]	Purposive sampling from urban community aged care services	Gender: 96% female<br>Age: 43.2 (9.7)<br>Employed: 7.9 (4.5) years	Content analysis
Goel (2015)[56]	Purposive sampling from 3 RACFs in a regional area	Gender: 100% female<br>Age: 30-55 years<br>Employed: 2-6 years	Thematic analysis
Gray (2017)[69]	Purposive sampling from four nursing facilities in single state in rural, suburban, and urban areas	Gender: 95% female<br>Age: 40.0 (9.5)<br>Employed: NR	Thematic analysis

Hoedl (2022)[71]	Purposive sampling from five urban and rural NHs	Gender: 78% female<br>Age: 41.0 (9.8)<br>Employed: 22% <5, 33% 5-10, 28% 11-20, 17% >20 years	Content analysis
Holmberg (2013)[66]	Purposive sampling from seven NHs in single state	Gender: NR<br>Age: NR<br>Employed: NR	Thematic analysis
Krein (2022)[67]	Purposive sampling from 13 facilities in single region	Gender: NR<br>Age: NR<br>Employed: 32% <1, 36% 1-5, 20% 6-10, 12% >11 years	Rapid analysis
Lim (2021)[59]	Convenience sampling from care worker support service, drawing on both home care and residential care workers	Gender: 100% female<br>Age: 59.0 (8.0) years<br>Employed: 7 (2.6) years	Content analysis
Marziali (2015)[84]	Purposive sampling from 17 rural and urban NHs	Gender: 92% female<br>Age: NR<br>Employed: 50% <5, 50% >5 years	Thematic analysis
Nichols (2015)[57]	Purposive sampling from 6 facilities in single region	Gender: NR<br>Age: NR<br>Employed: NR	Thematic analysis
Roussillon-Soyer (2021)[68]	Snowball technique to select seven NHs in one region	Gender: 95% female<br>Age: NR<br>Employed: 6.2 years	Thematic analysis
Senecal (2019)[86]	Convenience sampling from six SNFs in single state	Gender: 90% female<br>Age: 38.0 (12.0) years<br>Employed: 30% < 6 months, 20% < 1 year, 30% at least 1 year, 20% 2-10 years	Framework analysis
Sousa-Ribeiro (2022)[79]	Purposive sampling from single LTCF	Gender: 87% female<br>Age: 55-61 years<br>Employed: 6-38 years	Interpretative phenomenological analysis
Titley (2022)[74]	Convenience sampling from eight LTCFs across two regions	Gender: 94% female<br>Age: 26% <30-39, 35% 40-49, 24% 50-59, 14% >60 years<br>Employed: 10.3 (8) years	Content analysis
Winarnita (2022)[50]	Purposive sampling from known social networks, employees at 7 RACFs	Gender: 100% female<br>Age: 31-55 years<br>Employed: 1-10 years	Thematic analysis

ACA, aged care assistant; CA, care aide; CNA, certified nursing assistant; DCW, direct care workers; DON, director of nursing; LN, licensed nurse; HHA, home health aide; LTCF, long-term care facility; Mod, moderate; n/a, not applicable; NA, nurse assistant; NH, nursing home; NR, not reported; PCW, personal care worker; PSW, personal support worker; RACF, residential aged care facility; RN, registered nurse; SNF, skilled nursing facility; US, United States
