# Supplementary material for: Factors Impacting Retention of Aged Care Workers: A Systematic Review
Source: Healthcare (Basel). 2023 Nov 21;11(23):3008. doi: 10.3390/healthcare11233008 (PMC10706301; doi:10.3390/healthcare11233008)
Supplement: Supplementary file 1 [file healthcare-11-03008-s001.zip › Table S7_Synthesised findings.pdf]

Table S7. Synthesised findings of factors impacting care worker retention

Individual factors	Study Findings
Emotional health and wellbeing	Care workers report lower mental health scores than the general population [75], and feelings of abandonment, disrespect, burnout, and fear during the COVID-19 pandemic [76, 77]. Prioritizing personal well-being is difficult for care workers, especially those with responsibilities for others outside of the workplace [55, 64, 77]. Following a client death in home care or residents within facilities, care workers reported a desire for greater emotional support from their workplace [58, 59, 89]. Older care workers and those working in smaller facilities report greater mental health quality of life [52]. Targeted cognitive behavioral group training can improve employee mental health [47].
Satisfaction with work role	Higher job competency, salary [54, 70], personalized care provision, caring work climate, validation, feedback [54, 56, 57, 69, 71] and organizational support [50, 55, 72] are associated with satisfaction and intention to stay. Stress related to resident behavior [55, 59, 62], the physical nature of the role [50, 62, 69, 79] and lower supervisor support reported to reduce satisfaction [73]. Stress related to fear of infection (self or transmitting to others) during the COVID-19 pandemic reduced satisfaction in care worker staff [71, 74].
Care worker age	Retention of older workers positively influenced by work latitude, optimal utilization [80], and flexibility of casual employment [79]. Negatively influenced by limited possibilities to adapt work tasks or demands, with perceived threats to own health [79]. Younger care workers report higher rates of emotional abuse from resident families [83], and higher rates of intention to leave [85].
Identity related to work role	Negative feelings of stigmatization, lack of respect associated with complexity of care tasks or acknowledgement of efforts made to provide personalized care [59, 64-68]. Positive feelings towards providing a compassionate, nurturing, and multi-faceted service to vulnerable elderly [64, 66, 69] that is an intrinsic part of personality as "caregivers" [55, 66].
Troubled conscience	Care workers express concerns about own level of competence or making the "right' decision for residents [59, 67, 76, 89]. Group training exploring troubled conscience can improve work-related social support [46].
Staff supervision and management	Clinical leadership training can improve supervisor support [45, 60] and management behavior towards care workers [45]. Leadership styles found to positively affect professional employees more than care workers [93]. Greater leadership scores [81], loyalty towards care workers [94], responsive actions towards reports of discrimination [50], and low management turnover [81, 82] associated with higher care worker retention rates. Professionals and care workers agree that collaboration improves quality of care [88] and leadership should be accessible and responsive [77]. Lower levels of supervisor support associated with reduced care worker job satisfaction [73] and were found to be lacking during the COVID-19 pandemic [76].
Hierarchy of care roles	Low empowerment of care workers associated with higher turnover [59, 81, 82], lower job satisfaction [82] and lack of ability to influence care decisions [65, 84], with reports of disrespect from managers towards care workers [64, 66, 69, 86, 87]. In facilities with well-functioning teams, care workers report open communication and mutual respect across team members as positive factors [55, 84].
Workplace culture	Perceived lack of teamwork [62, 65, 85, 86], lack of appreciation [62], bullying or mistrust [65, 69] between care workers and across other professionals involved in care [56, 66, 77, 87], is associated with intention to leave [65, 85], and highlights power inequities between peers [56, 87]. In some instances, the COVID-19 pandemic raised team morale, cooperation, and communication [64, 71]. Socially supportive colleagues [74, 79] are reported to positively impact job satisfaction and intention to stay. Migrant workers reported experiencing discrimination and racism from residents, colleagues, and families [50, 55-57].

Relationship between care worker and families of residents	High reported rates of threatening behavior, bullying and emotional abuse from resident families towards care workers [83], with conflict management responsibility differing between supervisors or care workers across facilities [84]. Care workers report their knowledge or commitment to care being brought into question by resident's families [66].
Relationship between care worker and residents	Care workers describe strong attachments and genuine affection for residents [50, 64] with attraction to the role of caring [55, 65]. Despite expressing an intention to leave, care workers reported concern for resident wellbeing as a driver to stay [65]. Work-related loss/grief and conflict with residents increases stress amongst care workers [64].
**Organisational factors**	**Study findings**
Resources- staffing	Workplace stress related to perceived understaffing noted as the greatest challenge to care workers [74] and attributed in part to a lack of continuous, competent staff [76, 79] and exacerbated by onset of the COVID-19 pandemic [71, 74, 76]. Staff absences that are not covered place additional demands on care workers [56, 62, 66, 68, 77, 79, 86, 89], reduce opportunity for person-centered care [59, 67, 74, 87] and increase turnover [67]. Targeted cognitive behavioral group training can reduce staff absence [47]. Care workers expressed desire for schedule control [62], even if that resulted in monetary loss [68], with staff-empowered work teams finding absences less impactful [90].
Permanency of contracts, shift length, break allowances, and leave entitlements	Lowered job satisfaction associated with longer shift lengths, split shifts, insufficient break allowances [59, 64, 91], casual contracts [49, 50, 54, 56] and burnout syndromes [91]. Home care worker reports of vacation leave resulting in unemployment due to the perceived impact on the elderly clients who prefer continuity [59].
Workplace injury	High rates of injury amongst older, female care workers [78], those with lower social supports, migrant workers and those not using assistive devices for manual handling [53, 61], with an increased level of intention to leave [78].
On-the-job training, opportunities for enrichment or promotion	Lack of training or educational pathways associated with increased stress [56, 57, 59, 64, 69], with promotional opportunities related to increased retention [85], and a desire for increased responsibilities [86]. Care worker training was reported to be difficult to access financially, not relevant to the realities of work [59], and not provided to substitute staff [68]. Migrants with overseas nursing qualifications reported care worker roles enabled practical experience in new country before obtaining formal registration for nursing [50, 55, 57].
Wages	Small increases in wage not effective in reducing care worker turnover [48], with poor wages associated with increased stress [64] and low status of the role [79]. Care workers expressed belief that retention would improve if wage rises were associated with length of tenure [67] or contribution to organizations [68]. Care workers expressed frustration with unregulated wage system and attributed the quick turnover of new staff to the realization that wages were lower than other facilities [59].
Profit status of facility	For-profit facilities associated with high turnover [82]. Lower staffing ratios in for-profit facilities impact ability to provide person-centered care [65, 86].
Demands of role	Lower perceived work demands associated with lower work-related influence, migrant employees and working fixed evening or night shifts [51]. Heavy workloads associated with higher rates of intention to leave [85]. Limited scope to perform non-physical tasks influencing intention to stay amongst older care workers [79]. Care worker scope of practice reported to be uncertain, leading to exhaustion and burnout [59].
Job stability	Increased job stability associated with increased retention [55, 85] and reduced work-related stress [79]. Home care workers describe lower job stability than facility care workers due to personal preference of clients [59] and higher rates of casual employment contracts amongst migrant workers [49]. Overseas-qualified nurses reported working in lower-skilled roles such as personal care attendants due to non-recognition of overseas education [55, 57] and were more likely to work multiple jobs due to underemployment, especially when coming from a non-English speaking background [49, 54, 57].

Pre-employment training	Insufficient on-site training before starting role [62, 65, 67], with care workers desiring practical education in physical and emotional support for end-of-life care situations [58, 77, 89].
Location of facility	Regional areas reported difficulty with attracting care workers from urban locations [85], however migrant staff reported benefits of working in smaller communities with increased job security especially with peers from similar cultural backgrounds [50, 56].
Local unemployment rates	Low rates associated with difficulties in care worker retention as wages comparable in less stressful industries [62, 82].
Access to improved conditions in other aged care facilities	Higher retention found to be associated with the presence of a union [81] or in workplaces with lower proportions of residents with non-dementia psychiatric illnesses [82].
Pandemic related policies	Care workers usually working multiple part-time jobs reported significant financial implications of being limited to a single workplace [74]. Some care workers reported that visitor restrictions resulted in a calmer, relaxed workplace [71]. Others identified great stress and guilt at observing resident distress and deterioration in the absence of family [71, 74]. Restrictions on other health professionals e.g., activity coordinators, placed additional burden on care workers [71]. Care workers reported stress related to changing regulations, personal protective equipment requirements and best practice guidance [71, 74, 76]. Some care workers perceived quality of personal protective equipment provided was of a lower quality than that worn by visiting professionals [76].
Minimum ratios	Higher ratios of professional to care worker staff associated with increased supportive supervision. Low staff to resident ratios associated with higher rates of work-related injuries [61] and intention to leave [85].
