# Supplementary material for: Factors Impacting Retention of Aged Care Workers: A Systematic Review
Source: Healthcare (Basel). 2023 Nov 21;11(23):3008. doi: 10.3390/healthcare11233008 (PMC10706301; doi:10.3390/healthcare11233008)
Supplement: Supplementary file 1 [file healthcare-11-03008-s001.zip › Table S8_reasons for exclusion at full text review.pdf]

Supplementary table S8_Reasons for exclusion at full text review

Study	Title	Notes
Agosti 2015	Shift work to balance everyday life - a salutogenic nursing perspective in home help service in Sweden	Exclusion reason: Not including aged care support workers;
Ahlin 2013	Longitudinal relationships between stress of conscience and concepts of importance	Exclusion reason: Outcomes not related to recruitment or retention of aged care support workers;
Amateau 2022	Stress, strength, and respect: Viewing direct care staff experiences through a trauma-informed lens	duplicate
Assis 2020	Training Program for Care Professionals in Nursing Homes: Effectiveness and Feasibility	Exclusion reason: Outcomes not related to recruitment or retention of aged care support workers;
Backhouse 2022	Home-care workers' experiences of assisting people with dementia with their personal care: A qualitative interview study	Exclusion reason: Outcomes not related to recruitment or retention of aged care support workers;
Beck 2015	Applying a palliative care approach in residential care: effects on nurse assistants' work situation	Exclusion reason: Data from pre-2012;
Beck 2012	Having to focus on doing rather than being-nurse assistants' experience of palliative care in municipal residential care settings	Exclusion reason: Data from pre-2012;
Bissoondial 2014	Job satisfaction and its relationship on retention: A study of long-term care nurses in Ontario, Canada	Exclusion reason: Conference proceedings, dissertation;
Boerner 2015	Grief after patient death: direct care staff in nursing homes and homecare	Exclusion reason: Outcomes not related to recruitment or retention of aged care support workers;
Bolt 2020	Nursing Staff Needs in Providing Palliative Care for Persons With Dementia at Home or in Nursing Homes: A Survey	Exclusion reason: Data not separating aged care support worker outcomes;
Brady 2016	An exploration of nursing assistants' perceptions about job satisfaction	Exclusion reason: Non research paper
Butler 2012	Older Women Caring for Older Women: The Rewards and Challenges of the Home Care Aide Job	Exclusion reason: Data from pre-2012;
Carter 2013	Sleep disturbance, chronic stress, and depression in hospice nurses: testing the feasibility of an intervention	Exclusion reason: Not residential or home based aged care setting;
Champion 2017	What support do nurses need to provide palliative care for people with dementia?	Exclusion reason: Not residential or home based aged care setting;
Cheloni 2019	Working with older people with dementia: motivations and demotivations among health carers	Exclusion reason: Not residential or home based aged care setting;

Cheong 2021	Developing and Evaluating a Continuous Education Program for Healthcare Assistants in Macao: A Cluster-Randomized Trial	Exclusion reason: Outcomes not related to recruitment or retention of aged care support workers;
Choi 2021	Person-Centered Care Environment Associated With Care Staff Outcomes in Long-Term Care Facilities	Exclusion reason: Data not separating aged care support worker outcomes;
CouletduGard 2021	Perceptions of job factors that impact intent to leave among CNAS in LTC facilities in Delaware	Exclusion reason: Conference proceedings, dissertation;
Dhaini 2017	Are nursing home care workersâ€™ health and presenteeism associated with implicit rationing of care? A cross-sectional multi-site study	Exclusion reason: Outcomes not related to recruitment or retention of aged care support workers;
Dobbs 2018	Certified nursing assistants' perspectives of the CARES activities of daily living dementia care program	Exclusion reason: Outcomes not related to recruitment or retention of aged care support workers;
Dobbs 2018	Certified nursing assistants' perspectives of the CARES R activities of daily living dementia care program	Exclusion reason: Duplicate;
Dominguez 2022	Retention of nursing assistants in assisted living using stay interviews: A qualitative case study	Exclusion reason: Conference proceedings, dissertation;
Duan 2020	Care Aides Working Multiple Jobs: Considerations for Staffing Policies in Long-Term Care Homes During and After the COVID-19 Pandemic	Exclusion reason: Non research paper ;
Ewalt 2016	Is Empowerment Related to Certified Nursing Assistant Job Satisfaction? A Cross-Sectional Study	Exclusion reason: Full text not available;
Finn 2018	The Role of Empathy in Nursing Assistant Retention	Exclusion reason: Conference proceedings, dissertation;
Gallardo 2018	Personality predictors of compassion fatigue in long-term care environments	Exclusion reason: Data not separating aged care support worker outcomes;
Gosseries 2012	Burnout in healthcare workers managing chronic patients with disorders of consciousness	Exclusion reason: Not residential or home based aged care setting;
Hanson 2023	Comfort First: Development and pilot testing of a web-based video training to disseminate Comfort Matters dementia care	Exclusion reason: Outcomes not related to recruitment or retention of aged care support workers;
Harrad 2018	Factors associated with and impact of burnout in nursing and residential home care workers for the elderly	Exclusion reason: Non research paper ;
Hart 2020	Scheduling longer working lives for older aged care workers: A time and income capability approach	Exclusion reason: Data not separating aged care support worker outcomes;
Henry 2015	The effects of certified nurse assistants' socialization, onboarding, and turnover	Exclusion reason: Conference proceedings, dissertation;

Holmsen 2023	Professional caregivers' participation in the International Caregiver Development Programme: A qualitative study of psychosocial care in nursing homes	Exclusion reason: Outcomes not related to recruitment or retention of aged care support workers;
Hurtado 2012	Racial disparities in job strain among American and immigrant long-term care workers	Exclusion reason: Data from pre-2012;
Jungyoon 2014	The joint relationship between organizational design factors and HR practice factors on direct care workers' job satisfaction and turnover intent	Exclusion reason: Data from pre-2012;
Knoppâ€Sihota 2015	Factors associated with rushed and missed resident care in western Canadian nursing homes: a cross-sectional survey of health care aides	Exclusion reason: Data from pre-2012;
Ko 2012	Aggression Exhibited by Older Dementia Clients Toward Staff in Japanese Long-Term Care	Exclusion reason: Data from pre-2012;
Lachs 2012	The epidemiology of physically and verbally aggressive behaviors of nursing home residents directed at staff	Exclusion reason: Conference proceedings, dissertation;
Lange 2013	The ELDER expansion project: building cultural competence among long term home care workers	Exclusion reason: Outcomes not related to recruitment or retention of aged care support workers;
Lee 2013	Attitudes of long-term care staff toward dementia and their related factors	Exclusion reason: Data from pre-2012;
Long 2019	Exploring Certified Nurse Aide Perspectives on Caring for Residents With Dementia	Exclusion reason: Non research paper ;
Long 2018	Echo-chicago geriatrics SNF: A pilot direct care workforce education initiative	Exclusion reason: Conference proceedings, dissertation;
Lucchetti 2014	Nursing home care: Exploring the role of religiousness in the mental health, quality of life and stress of formal caregivers	Exclusion reason: Data from pre-2012;
Mager 2016	The ELDER Project	Exclusion reason: Data not separating aged care support worker outcomes;
Midje 2021	Exploring work engagement in the context of person-centred practices: a qualitative study in municipal long-term care facilities for older people	Exclusion reason: Data not separating aged care support worker outcomes;
Midje 2022	The role of working environment and employee engagement in person-centred processes for older adults in long-term care services	Exclusion reason: Data not separating aged care support worker outcomes
Miranda 2014	Musculoskeletal pain and reported workplace assault: a prospective study of clinical staff in nursing homes	Exclusion reason: Data not separating aged care support worker outcomes;

Mountford 2013	I'll take care of you: The use of supportive work practices to retain older workers	Exclusion reason: Non research paper ;
Novek 2013	Filipino Health Care Aides and the Nursing Home Labour Market in Winnipeg	Exclusion reason: Data from pre-2012;
OrrungWallin 2015	Job strain and stress of conscience among nurse assistants working in residential care	Exclusion reason: Data from pre-2012;
Pelissier 2015	Impaired mental well-being and psychosocial risk: a cross-sectional study in female nursing home direct staff	Exclusion reason: Data from pre-2012;
Phua 2023	Educational needs of nursing home staff: does a needs-based palliative care course make a difference?	Exclusion reason: Outcomes not related to recruitment or retention of aged care support workers;
Pinto 2021	The impact of Covid-19 on certified nursing assistants in New York City: A cross-sectional study	Exclusion reason: Conference proceedings, dissertation;
Quach 2021	Relationship Between Work Experience and Safety Climate in Veterans Affairs Nursing Homes Nationwide	Exclusion reason: Outcomes not related to recruitment or retention of aged care support workers;
Rashik 2023	Have Nursing Assistant Stressors Changed Following COVID- 19?	Exclusion reason: Conference proceedings, dissertation;
Rasmussen 2015	A multifaceted workplace intervention for low back pain in nurses' aides: a pragmatic stepped wedge cluster randomised controlled trial	Exclusion reason: Data not separating aged care support worker outcomes
Rodriguez 2013	A brief educational intervention to improve staff management of disruptive behaviors in the nursing home	Exclusion reason: Conference proceedings, dissertation;
Rodwell 2014	Addressing workplace violence among nurses who care for the elderly	Exclusion reason: Data not separating aged care support worker outcomes;
Sarabia-Cobo 2021	Burnout, Compassion Fatigue and Psychological Flexibility among Geriatric Nurses: A Multicenter Study in Spain	Exclusion reason: Data not separating aged care support worker outcomes;
Sarabia-Cobo 2021	Experiences of geriatric nurses in nursing home settings across four countries in the face of the COVID-19 pandemic	Exclusion reason: Data not separating aged care support worker outcomes;
Schenck 2019	Efforts and challenges in retaining staff in a rural nursing home	Exclusion reason: Conference proceedings, dissertation;
Schmidt 2013	Job demands and personal resources in their relations to indicators of job strain among nurses for older people	Exclusion reason: Data not separating aged care support worker outcomes;
Schulze 2022	Psychosocial burden in nurses working in nursing homes during the Covid-19 pandemic: a cross-sectional study with quantitative and qualitative data	Exclusion reason: Data not separating aged care support worker outcomes;
Schwendimann 2016	Factors associated with high job satisfaction among care workers in Swiss nursing homes - a cross sectional survey study	Exclusion reason: Data not separating aged care support worker outcomes;

Senecal 2020	State Tested Nursing Assistants' Workplace Experiences That Threaten or Support Intent to Stay	Exclusion reason: Duplicate;
Shin 2014	Nursing Staffing and Quality of Life in Western New York Nursing Homes	Exclusion reason: Outcomes not related to recruitment or retention of aged care support workers;
Snyder 2021	A qualitative assessment of factors affecting nursing home caregiving staff experiences during the COVID-19 pandemic	Exclusion reason: Data not separating aged care support worker outcomes;
Soyer 2021	The demotivating impact of absenteeism in nursing homes	Exclusion reason: Duplicate;
Stutte 2017	Factors associated with aggressive behavior between residents and staff in nursing homes	Exclusion reason: Data not separating aged care support worker outcomes;
Temkin-Greener 2020	Nursing Home Staff Turnover and Perceived Patient Safety Culture: Results from a National Survey	Exclusion reason: Outcomes not related to recruitment or retention of aged care support workers;
Trinkoff 2013	Turnover, staffing, skill mix, and resident outcomes in a national sample of US nursing homes	Exclusion reason: Data from pre-2012;
Tufte 2012	Client-related work tasks and meaning of work: Results from a longitudinal study among eldercare workers in Denmark	Exclusion reason: Data from pre-2012;
WaiNingKris 2014	Recruitment challenges facing elderly care service providers in Hong Kong	Exclusion reason: Data not separating aged care support worker outcomes;
Wang 2022	Understanding person-centered dementia care from the perspectives of frontline staff: Challenges, opportunities, and implications for countries with limited long-term care resources	Exclusion reason: Outcomes not related to recruitment or retention of aged care support workers;
Wang 2015	Nurses' Practice Environment and Their Job Satisfaction: A Study on Nurses Caring for Older Adults in Shanghai	Exclusion reason: Not including aged care support workers;
Yen-Patton 2012	Nurse and nursing assistant reciprocal caring in long term care: Outcomes of absenteeism, retention, turnover and quality of care	Exclusion reason: Conference proceedings, dissertation;
